# Neuroticism predicts national vaccination rates across 56 countries

**DOI:** 10.1007/s12144-023-04234-8

**Published:** 2023-01-21

**Authors:** Nicolas Vermeulen

**Affiliations:** Psychological Sciences Research Institute (IPSY), Université catholique de Louvain (UCLouvain), Louvain-la-Neuve, Belgium and Fund for Scientific Research (FRS-FNRS), Brussels, Belgium

## Abstract

Quite strikingly, there is significant variation in Covid-19 vaccine coverage around the world. Some countries do not progress from around 2-3% while others are close to 100% coverage. In addition to some already known economic, health and sociodemographic predictors, the present research is interested in emotional factors that may predict a significant part of this cross-country variation. We examined the personality factor Neuroticism, which corresponds to the relatively stable tendency to experience negative emotions, anxiety and low tolerance for stress. Results confirm that gross domestic product represents around 50 percent of cross-country variation. Neuroticism added 6 to 9 percent of inter-country variation in vaccination coverage. The results are discussed in relation to the associations between Neuroticism, increased worry, greater attention to Covid-19 related information and confidence, as well as lower vaccine hesitancy.

From early 2020, humans around the world have been threatened to varying degrees by the emergence of a new Coronavirus (World Health Organization, 2020). The resulting fear and anxiety have subsequently shaped human thinking and behavior, both individually and collectively (Sobkow et al., 2020; da Silva Castanheira et al., 2021; Foad et al., 2021; Pazhoohi & Kingstone, 2021).

Initially, the pandemic was controlled by non-pharmacological interventions (NPIs) like masking or containments, while many researchers argued that NPIs had either little effect (Bendavid et al., 2021) or that policymakers were unable to weigh up costs and benefits of these NPIs properly (Lewis, 2022). One year later, from early 2021, the world has access to vaccines, but national coverage rates vary widely from country to country. One year after the launch of the Covid-19 vaccines, we can observe on the website Ourworldindata.org (January 31, 2022) that a country like Ethiopia plateaued at less than 2% of complete vaccination, Romania at 28%. The main industrialized nations (e.g., France, Germany, Israel, UK, USA) are in the middle of the table (around 55-75%) of this international ranking of vaccination rates, which is led by Chile and Portugal, which approach 90-100%.

Given that these vaccination campaigns address a health threat, individual differences such as threat sensitivity may help to account for variability in vaccination rates across countries. We examined the well-validated Neuroticism personality factor (McCrae & Costa, 1987; Lahey, 2009), which is the tendency to experience negative emotions, such as anxiety, worries and is associated with low tolerance for stress or aversive stimuli (McCrae & Costa, 1987). People who score high in neuroticism are more prone to understand everyday situations as threatening. Neuroticism, which is directly related to emotional reactivity and sensitivity to threat, appears to be of prime importance for the adoption of a strategy for dealing with a large-scale lethal threat. Neuroticism has been proposed as being important for public health (Lahey, 2009), and, some recent papers have shown that neuroticism positively predicts Covid-19 vaccine acceptance in the UK general population (Halstead et al., 2022) as well as the stability of intention (i.e., attitudes) toward recommended standard vaccinations in German patients with multiple sclerosis (Heidler et al., 2022). We hypothesized that this factor represents an excellent candidate to predict a significant part of variability in vaccination rates across countries.

## Method

We selected the Neuroticism levels across 56 countries (Schmitt et al., 2007) and three of the main vaccination indicators (per 100 inhabitants) available in ourworldindata.org (OWID) as of 31 January 2022 (See Table 1): people vaccinated per hundred (at least one vaccine dose); people fully vaccinated per hundred (all prescribed doses); total boosters per hundred (additional doses). OWID is a centralized inventory of official national data published on each government platform. More information can be found here https://ourworldindata.org/coronavirus-source-data. We also entered other factors that have been shown to predict Covid-19 vaccination coverage, namely gross domestic product per capita, the median age of a country’s citizens and the rate of obesity (Body Mass Index, BMI rates >30), population size and population density (Basak et al., 2022; Oshakbayev et al., 2022). We hypothesized that neuroticism plays a significant positive role in predicting cross-country variability in vaccination rates, beyond the effects of the other health predictors. The study complies with the 1964 Declaration of Helsinki and its later addenda and an informed consent was sought and obtained from participants when necessary.

**Table 1:** Data concerning the 56 countries involved in the analyses. Selected on January 31, 2022 from Ouwourldindata.org (OWID) and for Neuroticism from Schmitt et al. (2007)

Country code	Country name	Continent	Neuroticism standardized T-scores (Schmitt et al., 2007)	People vaccinated_At least one dose _OWID (%)	People Fully vaccinated_ _OWID (%)	Total Boosters (%) OWID	OBESITY (%) BMI > 30 (OWID Year 2016)	Median age Year (OWID)	Gross Domestic Product per capita Million Dollars (OWID)
ARG	Argentina	South America	55,05	86,88	76,11	27,2	28,3	31,9	18,933,907
AUS	Australia	Oceania	50,82	83,95	78,31	30,11	29	37,9	44,648,71
AUT	Austria	Europe	49,69	74,69	74,66	48,94	20,1	44,4	45,436,686
BGD	Bangladesh	Asia	51,2	57,39	35,91	0,76	3,6	27,5	3523,984
BEL	Belgium	Europe	53,6	78,62	76,26	56,33	22,1	41,8	42,658,576
BOL	Bolivia	South America	50,29	56,63	44,06	7,33	20,2	25,4	6885,829
BWA	Botswana	Africa	48,61	49,8	45,01	0	18,9	25,8	15,807,374
BRA	Brazil	South America	53,14	79,38	70	21,81	22,1	33,5	14,103,452
CAN	Canada	North America	50,58	84,95	79,07	39,81	29,4	41,4	44,017,591
CHL	Chile	South America	51,39	91,64	88,15	65,89	28	35,4	22,767,037
HRV	Croatia	Europe	44,58	56,16	53,96	14,57	24,4	44	22,669,797
CYP	Cyprus	Europe	46,16	73,65	70,23	45,12	21,8	37,3	32,415,132
CZE	Czech Republic	Europe	51,44	64,56	63,22	34,75	26	43,3	32,605,906
COD	Democratic Republic of Congo	Africa	51,02	0,4	0,23	0	6,7	17	808,133
EST	Estonia	Europe	46,99	64,47	62,63	0	21,2	42,7	29,481,252
ETH	Ethiopia	Africa	46,12	7,95	1,36	0	4,5	19,8	1729,927
FJI	Fiji	Oceania	48,03	73,65	68,02	0	30,2	28,6	8702,975
FIN	Finland	Europe	47,84	77,46	74,5	44,6	22,2	42,8	40,585,721
FRA	France	Europe	52,29	79,77	76,32	48,06	21,6	42	38,605,671
DEU	Germany	Europe	50,29	75,09	73,3	52,27	22,3	46,6	45,229,245
GRC	Greece	Europe	53,19	74,71	70,28	46,25	24,9	45,3	24,574,382
HKG	Hong Kong	Asia	52,41	70,92	63,64	12,59	*missing*	44,8	56,054,92
IND	India	Asia	50	67,51	50,62	0,8	3,9	28,2	6426,674
IDN	Indonesia	Asia	49,73	65,95	45,38	0	6,9	29,3	11,188,744
ISR	Israel	Asia	49,27	72,01	65,54	54,63	26,1	30,6	33,132,32
ITA	Italy	Europe	51,66	83,09	76,29	54,91	19,9	47,9	35,220,084
JPN	Japan	Asia	57,87	80,48	79,14	3,24	4,3	48,2	39,002,223
JOR	Jordan	Asia	49,86	44,49	40,76	0	35,5	23,2	8337,49
LVA	Latvia	Europe	51,11	71,63	68,95	23,78	23,6	43,9	25,063,846
LBN	Lebanon	Asia	53,35	35,95	29,68	6,48	32	31,1	13,367,565
LTU	Lithuania	Europe	51,87	72,19	69,16	32,83	26,3	43,5	29,524,265
MYS	Malaysia	Asia	48,14	79,54	78,45	36,28	15,6	29,9	26,808,164
MLT	Malta	Europe	52,35	89,99	87,43	64,15	28,9	42,4	36,513,323
MEX	Mexico	North America	48	64,19	59,21	0	28,9	29,3	17,336,469
MAR	Morocco	Africa	50,87	66,28	62,05	0	26,1	29,6	7485,013
NLD	Netherlands	Europe	48,61	77,97	71,71	48,5	20,4	43,2	48,472,545
NZL	New Zealand	Oceania	49,59	81,29	76,55	26,11	30,8	37,9	36,085,843
PER	Peru	South America	53,39	75,1	68,5	23,41	19,7	29,1	12,236,706
PHL	Philippines	Asia	51,41	53,15	53,15	6,76	6,4	25,2	7599,188
POL	Poland	Europe	51,8	59,03	57,49	26,99	23,1	41,8	27,216,445
PRT	Portugal	Europe	50,21	94,15	90,47	49,14	20,8	46,2	27,936,896
ROU	Romania	Europe	48,03	28,62	27,91	0	22,5	43	23,313,199
SRB	Serbia	Europe	50,17	48,51	47,21	26,49	21,5	41,2	14,048,881
SVK	Slovak Republic	Europe	51,57	51,35	48,83	27,2	20,5	41,2	30,155,152
SVN	Slovenia	Europe	45,28	60,66	58,21	28,29	20,2	44,5	31,400,84
ZAF	South Africa	Africa	49,01	32,6	27,63	0,96	28,3	27,3	12,294,876
KOR	South Korea	Asia	53,99	87,01	85,75	53,04	4,7	43,4	35,938,374
ESP	Spain	Europe	54,03	87,52	81,91	45,98	23,8	45,5	34,272,36
CHE	Switzerland	Europe	48,72	69,64	67,92	39,07	19,5	43,1	57,410,166
TWN	Taiwan	Asia	53,13	80,03	73,12	22,85	25,1	42,2	*missing*
TZA	Tanzania	Africa	47,73	4,14	2,79	0	8,4	17,7	2683,304
TUR	Turkey	Asia	49,88	67,51	61,59	37,62	32,1	31,6	25,129,341
UKR	Ukraine	Europe	48,02	35,25	33,67	0	24,1	41,4	7894,393
GBR	United Kingdom	Europe	51,39	76,74	70,98	54,69	27,8	40,8	39,753,244
USA	United States	North America	50	75,12	63,53	26,39	36,2	38,3	54,225,446
ZWE	Zimbabwe	Africa	48,26	28,28	21,84	0	15,5	19,6	1899,775

## Results

Correlations with Neuroticism were highly significant for two of the three indicators (at least one dose: *r*(56) = .395, *p* = .003; fully vaccinated: *r*(56) = .376, *p* = .003), while the association with boosters remained marginal, *r*(56) = .238, *p* = .08. Regarding health predictors, the three vaccination rates were indeed significantly associated with variables such as gross domestic product per capita (.626 < *r* < .677, *p* < .001), the median age of the country’s citizens (.592 < *r* < .611, *p* < .001), and the rate of obesity (.278 < *r* < .342, *p* < .05). Population size or density did not correlate with vaccination rates. Importantly, partial correlations between neuroticism and vaccination rates remained significant after controlling for gross domestic product, median age, as well as for obesity rates (BMI >30) (.378 < *r* < .384, *p* < .01).

Stepwise regression analyses (Table 2) were then performed by first entering the gross domestic product, then the median age and the obesity rate (BMI >30) in a second step, and in the final step the average neuroticism level per country. The results of these regressions showed that the primary predictor of vaccination in a country was the country’s gross domestic product for all three vaccine indicators. Country gross domestic product alone represented between 41% and 53% of the cross-country variability in vaccination rates (R^2^ change). Neuroticism was the only other significant predictor for vaccination rate (at least one dose) and represented an additional 9% (R^2^ change) of the cross-country variance. The richer the country and the higher its average neuroticism score, the higher its rate of vaccination (at least one dose). In addition to gross domestic product, for the fully vaccinated rate, median age significantly predicts 5% of the additional variance while neuroticism adds significantly another 6% to the final model. The booster rate was only significantly predicted by gross domestic product, with neuroticism as well as any other predictors having no significant influence.

**Table 2:** Third step of the hierarchical stepwise regressions including remaining significant predictors of vaccination rates across 56 countries

	*R*^*2*^ Change STEP 3^*b*^	Standardized* β*	*t*	*p* value	
People vaccinated per hundred (at least one vaccine dose) – Step 3 ^a^					
Gross Domestic Product	.41***	.598	5.970	.001***	
Neuroticism	.09*	.303	3.026	.003**	
People fully vaccinated per hundred (all prescribed doses)- Step 3					
Gross Domestic Product	.48***	.44***	3.151	.001***	
Median age (year)	.05*	.29*	2.007	.05*	
Neuroticism	.06**	.25**	2.687	.01**	
Boosters per hundred (additional doses)					
Gross Domestic Product	.53*	.728	7.664	.001***	

Vaccination rates as well as gross domestic product, median age, Obesity rates (Body Mass Index >30) were extracted from Ourwourldindata.org on January 31, 2022

a. in a first step Gross Domestic Product per capita was entered. In a second step median age, Obesity rates (Body Mass Index >30). In a third step, Neuroticism was entered in the model

b. accounted additional variation of (*R*^*2*^ change) vaccination rates following the inclusion of Neuroticism in the third Step

* *p* < .05; **p < .01, ***p < .001

## Discussion

These findings highlight that neuroticism significantly predicts variations in country vaccination rates in different continents. These findings remained significant even after controlling for gross domestic product or physical health/risk factors such as age or obesity known to impact vaccine distribution (Basak et al., 2022; Oshakbayev et al., 2022). These results can be related at a more local level to data showing that neuroticism predicted negative affect level and variability during the COVID-19 pandemic in Germany as well as with neuroticism’s associations with mean levels of attention to COVID-19 related information and worry (crisis preoccupation) (Kroencke et al., 2020). If people scoring high on neuroticism paid more attention to COVID-19 related information and worried more about one’s own health during the pandemic, it may explain neuroticism’s role in adhering to a vaccines’ national strategy. Our results also relate to UK findings showing that lower levels of neuroticism are associated with increased COVID-19 vaccination hesitancy (Halstead et al., 2022).

However, our data, in combination with the above-mentioned results, may seem to contradict other data from Russia showing that neuroticism is positively associated with vaccine hesitancy or resistance (Roshchina et al., 2022). It should be noted that most of the above-mentioned literature is related to vaccine intention (or attitude) when vaccination campaigns had not yet begun. This differs notably from the present data, which relates to actual vaccination rates rather than to anticipated behavior (intentions). The contradiction could, for example, be related to trust in vaccines or in the government, given that a recent study showed that a significant portion (around 30%) of those who had been vaccinated had nevertheless a low level of confidence in the Covid-19 vaccines (Gbenonsi et al., 2022).

From this perspective, it is interesting to note that in Roshchina et al. (2022), a high level of trust in institutions was associated with a stronger intention to be vaccined. Confidence in the solution (i.e., vaccines) together with fear of Covid-19 may appear as key factors to account for the apparent contradiction. The “appeal to fear” model (Witte, 1992) emphasizes that for a promoted behavior to be considered, one must feel at risk (susceptibility), and one must be confident that the solution (or response) is effective, easy to implement, and cost-effective. The appeal to fear model predicts that if risk susceptibility is high but confidence in the solution is low, there might be denial of the threat and rejection of the solution. In this sense, it is not surprising to observe in Roshchina et al. (2022) that vaccine resistance and hesitancy were greater in the regions with worse epidemiologic situations, where fear is potentially stronger because of threat proximity. This proposed explanation fits quite well with data showing that anxiety during covid Covid-19 is negatively related to confidence, controllability and physical distance to the nearest Covid-19 covid patients (Wu et al., 2021). In this way, it might be suggested that when there is a threat and confidence in the solution is high, neuroticism could increase acceptance, whereas if confidence is low, neuroticism could increase hesitancy.

Future research could aim to better understand how neuroticism not only influences vaccine intention but also the transformation of these intentions into actual behaviors. One way might be to use the photovoice method, which allows researchers to understand facilitators and barriers from the unique perspective of each participant, to understand the contextual conditions of participants, and to increase social justice by adequately informing mental health service providers and policy makers (Tanhan & Strack, 2020).
